# Review of Topical Sodium Heparin 1000 IU/g Gel in Symptomatic Uncomplicated Superficial Thrombophlebitis

**DOI:** 10.7759/cureus.47418

**Published:** 2023-10-21

**Authors:** Francesc Cabré, Jairo A Camacho, Carlos A Rodríguez-Garcés, Débora V Breier, Martín Ballarin

**Affiliations:** 1 Medical Department, Menarini Group, Badalona, ESP; 2 Biochemistry and Molecular Biomedicine, University of Barcelona, Barcelona, ESP; 3 Medical Department, Menarini Group, Bogotá, COL; 4 General Medicine, General and Peripheral Vascular Surgery, Centro Médico La Sabana, Bogotá, COL

**Keywords:** antithrombotics, superficial thrombophlebitis, superficial vein thrombosis, topical heparin, heparin gel

## Abstract

Heparin, a mixture of sulfated polymorphic polysaccharides (glycosaminoglycan) chains of variable lengths and weights and a natural anticoagulant, is widely used in medical practice to prevent intravascular blood coagulation. Heparin has demonstrated antithrombotic and anti-inflammatory activity, and it is mostly administered systemically (intravenously or subcutaneously) for primary or secondary prevention of venous thromboembolism after surgical interventions, or immobilized patients, or on short-term antithrombotic therapy of patients with atrial fibrillation who must undergo treatment. However, since systemic administration of heparin could be, in certain cases, linked to an increased risk of bleeding, topical heparin is widely used for the prevention and treatment of local symptoms of peripheral vascular disorders, such as venous insufficiency, varicose veins, or superficial thrombophlebitis. This review summarizes the main safety and efficacy characteristics of the topical formulation of Heparin in Gel form (1000 International Units of Heparin/g Gel) currently in use, which has demonstrated an excellent efficacy and tolerability profile in reducing signs and symptoms of peripheral vascular disease, e.g., varicose syndromes and their complications, phlebothrombosis, thrombophlebitis, superficial periphlebitis, varicose ulcers, for post-operative varicophlebitis, sequelae of saphenectomy, for traumas and contusions, local edemas and infiltrates, subcutaneous hematoma and for traumatic affections of musculotendinous and capsuloligamentous apparatuses.

## Introduction and background

Sodium heparins are a heterogeneous group of linear chain anionic mucopolysaccharides with well-known anticoagulant properties. However, its phlebological indications in many countries mainly include varicose veins, superficial thrombophlebitis and periphlebitis, as well as postoperative cases. Topical sodium heparin's main effect on uncomplicated superficial thrombophlebitis or superficial vein thrombosis is anti-inflammatory. Venous disease, including varicose veins and chronic venous insufficiency, is one of the most frequently reported chronic conditions and a substantial source of morbidity [1-3]. Estimates of the incidence of venous disease vary widely by anatomic region and classification of pathology [4]. Topical sodium heparin is widely used for reducing the risk and treating local symptoms associated with peripheral vascular disorders [5]. However, despite its widespread use, few published data refer to the efficacy and safety of topical heparin. As safety is concerned, published reports consider topical heparin to be a pharmaceutical form of the drug with an acceptable safety profile [6].

The purpose of this review is to summarize different literature publications addressing the use of topical heparin sodium in the pharmaceutical form of gel to treat uncomplicated superficial thrombophlebitis, applied to patients, in relation to the improvement or resolution of signs and symptoms. A secondary objective is to identify, within the effective treatments, the characteristics of their application: frequency, dosage, etc.

## Review

Chronic venous disorders: Superficial thrombophlebitis

Venous disease is a very broad term whose definition can include both venous thromboembolism and chronic venous disease. Although there is some similarity in origin, the pathogenesis of these pathologies is quite different. Venous thromboembolism primarily manifests as deep vein thrombosis and/or pulmonary embolism caused by dysregulated coagulation, and its diagnosis is generally event-based. Chronic venous disease has been postulated to be the result of tissue damage due to prolonged venous hypertension, as a consequence of chronic venous insufficiency due to primary venous reflux or other causes of increased central venous pressure, such as obesity, prolonged standing, pregnancy, and other situations that cause vascular compression (Table 1).

**Table 1:** Summary of the most relevant clinical studies conducted with Heparin 1000 IU/g Gel (Lioton®/Menaven®). In most cases, clinical parameters were assessed according to a score scale. ALT: Alanine transaminase; AST: Aspartate aminotransferase; CRP: C-reactive protein; CVI: Chronic venous disease; ESR: Erythrocyte sedimentation rate; FDP: Fibrin degradation products; GGT: Gamma-glutamyl transferase; HDL-C: Cholesterol of high-density lipoproteins; IU: International Units; LDL-C: Cholesterol of low-density lipoproteins; NR: No reported; PA: Prothrombin activity; PC: Phosphatidylcholine; PT: Prothrombin time; PPT: Partial thromboplastin partial time; TG. Triglycerides; VLDL-C: Cholesterol of very low-density lipoproteins; PTT: Partial thromboplastin time.

Study	Patients diagnostic	Treatment duration	Evaluation	Efficacy and safety
D’Amico et al. [7]	Thirty patients: 17 CVI simple; seven CVI complicated; five superficial thrombophlebitis; and one postphlebitic syndrome.	Two gel preparations. (1) 100 g heparin sodium 100,000 IU. (2) aescin 1 g, heparin sodium 10,000 IU and PC. 0.8 g; 3-10 cm gel; 20 days.	Edema: spontaneous and induced pain, erythema: functional limitation, hematoma: sensation of heaviness, ulcers: paresthesias, nocturnal cramps: cutaneous dyscrasias, induration: vein turgor. Laboratory parameters: coagulation time, PTT, PT, ESR, red blood cells, hematocrit, hemoglobin, white blood cells, platelets, CRP.	Improving the symptoms examined (p<0.05) in favor of heparin-only preparation (edema, erythema, hematoma, induced pain, cutaneous dyscrasias). After seven to 10 days of treatment: excellent tolerated. No local or general allergic reactions were recorded.
Inga et al. [8]	Forty patients: 22 phlebitis and superficial periphebitis; 10 post-phlebitic syndrome; eight varicose ulcers.	(1) Test drug (20) heparin 1000 IU/g. (2) Reference drug (20) heparin 10.000 IU, aescine and PC. Average 57.5 days.	Edema: spontaneous and induced pain, erythema: functional limitation, hematoma: heaviness, ulcerations: paresthesias, Nigh cramps: cutaneous dyscrasias. Induration and turgor of vein. Laboratory parameters: coagulation time, PTT, ESR, leukocytes, platelets	Improvement in 85% of patients. Significant reduction (-26.7%) of ESR. None of the coagulation parameters was modified.
Spigonardo et al. [9]	Seventy-two patients: venous telangiectasia of lower limbs.	(1) Lioton® 1000 Gel vs (2) sulfuric polyester of a mucopolysaccharide (ref drug). Four weeks; two per day.	Hematoma. Hyperemia in the site of sclerosing therapy. Local edema.	Clear improvement with heparin mainly for the effects of regression of hematoma and hyperemia in comparison to the control. All patients of the heparin group had good acceptance. 11% (4/36) reference group showed pruritus and reddening of the skin.
Ungar [10]	60 (2 x 30) patients: Superficial phlebitis of the leg.	(1) Lioton® 1000 Gel (1000 IU/g); three per day; 10 cm; 850 IU. (2) Placebo. Additional antibiotic covering treatment, 14 days.	Erythema: functional limitation. Edema: paresthesias. Itching: sense of heaviness. Tumefaction of the vein. Spontaneous and induced pain. Laboratory parameters: erythrocytes, leukocytes, hematocrit, hemoglobin, ESR, GGT, AST, ALT, bilirubinemia, azotemia, glycemia, creatininemia, PT, PTT.	Significant improvement in the Lioton® group vs placebo in all parameters except sense of heaviness. No significant change on coagulation parameters in any of the two groups. In the Lioton® group statistically significant variations on white-blood count and the ESR. Lioton®: one case of excessive skin dryness and one case of erythema.
Pola et al. [11]	Twenty patients: Varicose phlebitis of the lower limbs, of recent onset, and of an extent limited from 5 to 15 cm.	Lioton® 1000 Gel (1000 IU/g), 5-15 cm gel (1245-3735 IU). Three/day + 830-2490 IU two/day. 15 + 15 days.	Cutaneous tolerance (pain, erythema, and edema). Laboratory parameters: blood and plasma viscosity, HDL-C, LDL-C, VLDL-C, TG, PTT, and PT.	Improvement in the objective and subjective symptomatology. No side effects of local or systemic. No significant changes in the laboratory parameters.
Capelli [12]	Twenty patients: six varices; 14 phlebitis and periphlebitis of lower and upper limbs.	Lioton® 1000 Gel (1000 IU/g). 5-10 cm gel. Two to three/day. Three to 20 days.	Edema, erythema, cutaneous dyscrasia, induration and turgor of veins, and ulcers. Laboratory parameters: coagulation time, PTT, ESR, erythrocytes, hematocrit, hemoglobin, white corpuscle, platelets, CRP, urinalysis, and FDP.	Good response in 85% of the cases. Anti-inflammatory activity and good absorption. After two weeks some symptoms of varicose syndrome decreased. No concerns, no tolerance.
Milio et al. [13]	Thirty patients. Phlebopathy with a phlogistic imprint of the lower limb: 16 varicose phlebitis; 11 superficial thrombophlebitis; three periphlebitis.	Lioton® 1000 Gel (1000 IU/g): three/day, 18-25 days.	Edema: spontaneous and induced pain, erythema: functional limitation, sense of heaviness, ulcerations, paresthesia, night cramps, skin dyscrasia, induration and turgor of the veins. Plethysmography and speedometric control of the lower limbs. Laboratory parameters: coagulation time, PTT, PT, platelets, ESR, and leukocytes.	Statistically significant improvement of most of the clinical parameters. Particularly pain and edema which exceptions night cramps and cutaneous dyscrasias. Normalization of ESR and leukocytes. No change in blood clotting parameters. No significant modification in plethysmographic and speedometric parameters.
Colonna et al. [14]	Eight hundred ninety-one hospitalized patients (P-IV study): 20% thrombophlebitis; 19% varices; 12% venous insufficiency; 10% post-saphenectomy; 8% dermatocellulitis; 8% subcutaneous hematomas; 7% varicophlebitis; 5% local traumas; 13% "other disorders".	Lioton® 1000 Gel (1000 IU/g). one to three/day. At least two weeks. Average 25.2 days.	Pain at rest and on movement. Edema: functional capacity, sense of limb heaviness, erythema, paresthesia, skin lesions, adverse reactions. Laboratory parameters: coagulation time, PTT, PT, platelets.	Significant improvement at the end of treatment: edema (53.9% vs 6.1%); spontaneous pain (54.3% vs 3.1%); individual pain (73.3% vs 8.8%); sense of heaviness (68.2% vs 10.4%), and functional capacity (39.1% vs 5.0%). Thirty-five patients with side effects: pruritis (10); erythema (6); allergy (5); dermatitis (5); dry skin (4); burning (2); sense of cold (2); urticaria (1). 18 (2%) patients withdrew due to unwanted effects. Slight significant increase on completion of PTT.
Navratilova and Semradova [15]	Thirty-two patients: (30 in assessment of efficacy): 15 superficial phlebitis; 12 hypodermitis; four complications after sclerotherapy.	Lioton® 1000 Gel (1000 IU/g). Two times/day, 3-10 cm length, four weeks.	Induration, pain, swelling, impairment of extremity function.	Percentage success rate of the therapy: 80% induration; 88.9% pain; 93.8% impairment of function; 85.7% edema.
Bihari [16]	Four hundred twelve patients. Superficial phlebitis.	Lioton® 1000 Gel (1000 IU/g). Area 2 cm wide and max 10 cm long (<20 cm^2^). The study did not include a control group. Two weeks.	Spontaneous pain. Pain on palpation or pressure. Ankle edema. Extent of skin rash. Size and number of thrombophlebitic fascicles or nodules. Elevated body temperature. Satisfaction of the treating physicians	Superficial phlebitis was improved in 96% of patients treated with Heparin Gel. Patients answers: healed or asymptomatic status: 124 (30.0%); improvement and treatment continuation: 272 (66.0); ineffective treatment: 5 (1.2%); phlebotomy or scheduled phlebotomy: 5 (1.2%); side effects (skin rash, burning sensation): 4 (1.0%); hospitalized for worsening of general condition 2 (0.5%).
Vilardell et al. [17]	132 (2 x 66) patients. Superficial phlebitis is secondary to indwelling intravenous catheters.	(1) Lioton® 1000 Gel (1000 IU/g); three/day. (2) Placebo. Max seven days.	Disappearance of the symptoms and signs of superficial phlebitis.	The clinical course of superficial phlebitis was similar in both groups. Investigator impression is favorable in the Lioton® group. End of the follow-up period phlebitis was rated as “severe” in 1/59 patients of Lioton® group and in 6/62 in placebo. Only one adverse event: mild contact urticaria. Investigator impression: tolerability good in 92.3% placebo and 86.9% topical heparin.
Velluti et al. [18]	Thirty-three patients. Athletes of different sports activities with direct or indirect traumatic lesions (contusions, hematomas, tendinitis, sprains).	Lioton® 1000 Gel (1000 IU/g). Three/day. Five to 10 days.	Pain (spontaneous and induced). Tumefaction. Functional limitation. Possible local relapses and duration of clinical recovery.	No complaints about local or general tolerance. The symptomatology was progressively reduced and on completion of the treatment, the indexes appeared to be favorable with no exceptions.
Virgilio and Cavallaro [19]	Seventy-one patients: 12 varicose phlebitis; 12 phlebitic thrombosis; 16 superficial thrombophlebitis; 21 post-saphenectomy hematoma; 10 varices	Lioton® 1000 Gel (1000 IU/g). Three/day (1500 – 5000 IU). Seven to 28 days.	Edema, erythema, functional limitation, hematoma, pain, sense of heaviness, itching. Laboratory parameters: PTT, PT, PA, fibrinogen, erythrocytes, hemoglobin, platelets, white corpuscles.	Noticeable and prompt improvement in the subjective and objective symptomatology. Complete regression of symptomatology on completion of treatment. When only Heparin Gel is administered no significant changes in laboratory parameters. One case of dermatitis due to allergy to the gel.
Colombo et al. [20]	Twenty-five patients. Professional footballers with local bruising injuries, mainly to the lower limbs.	Lioton® 1000 Gel (1000 IU/g). Two to three/day. 310 cm per application (900-3000 IU). Average 7.4 days.	Spontaneous and induced pain, edema, functional limitation, hematoma, feeling of heaviness, hardening, erythema. Laboratory parameters: Coagulation time, PTT, erythrocytes, leucocytes, platelets.	Rapid therapeutic response in all patients without any change in the laboratory parameters. Erythema is not significant because of the small number of cases treated.
Marrapodi et al. [21]	Twenty patients: six CVI post-phlebitic disease; seven CVI due to varices; two primary CVI; five acute superficial thrombophlebitis; 9/20 with venous ulcerations.	Lioton® 1000 Gel (1000 IU/g). 249 IU per cm of gel. Once a day. Max 30 days.	Sense of heaviness, paresthesia, pruritus, night cramps, edema, turgor of veins, hematoma, erythema, spontaneous and induced pain, cutaneous induration, skin dyscrasias, panniculitis, liponecrosis, functional limitation, Doppler speedometric and reflexed-light rheography venous examination. Laboratory parameters: platelets, PTT, clotting time.	In de treated group all the parameters appeared to be noteworthy improved. Statistically significance on day 15 of treatment for all signs and symptoms, except paresthesia, night cramps, and turgor of vein, improved on completion. Favorable evolution of ulcerations. Efficacy (physician judgment): excellent 60, good 30%. Two patients showed topical adverse effects (erythema and dryness of skin). Acceptance (physician judgment) excellent 70, good 25%. No significant changes in the main blood-clotting.
Daróczy [22]	One hundred and forty-seven patients. Lower limb varicosity syndrome.	Lioton® 1000 Gel (100,000 IU/100 g). Three times/day, 14 days.	Spontaneous and pressure pain in the region of varicosity. Calf circumference. Appearance of muscle spasm (calf muscle cramps). Heavy leg.	Spontaneous pain decreased second visit and significantly third visit. Tenderness decreased significantly, but less extent. Calf circumference improved. Calf muscle cramps decreased. Nocturnal cramps decreased to a lesser extent. Heavy leg improved at the end of the study. Satisfaction of patients: 88% wanted to continue treatment and 1% didn’t give an answer.

Chronic venous disease develops over a longer period of time (months to decades) and has a variety of clinical manifestations, including varicose veins, swelling, skin changes, and venous leg ulcers, which are often associated with significant discomfort [23,24]. Telangiectasia, reticular venectasia, varicose veins, and venous insufficiencies ranging from edema to active skin ulcers represent the most common clinically visible manifestations of chronic venous disease. As regards chronic venous insufficiency, it would be related to a wide spectrum of both structural and functional pathologies of the venous system [23,24].

Superficial thrombophlebitis or superficial vein thrombosis is a frequent pathology associated with chronic venous disease and a relatively common inflammatory thrombotic disorder that involves the development of a thrombus in a vein located near the surface of the skin. Signs and symptoms of this condition include pain, redness, warmth, and tenderness in an area along the affected vein, as well as erythema and swelling in surrounding tissue [25].

As a specific form of thrombophlebitis, chronic lymphovenous insufficiency is characterized by periodic exacerbations of the process in the form of acute superficial venous thrombophlebitis, erysipeloid inflammation, or deep vein thrombosis with the development of post-thrombophlebitic syndrome [26]. For example, in the clinical study CALISTO, almost 90% of patients were symptomatic and acute [27]. Superficial vein thrombosis is related to one of the components of Virchow's triad: intimal damage, stasis or turbulent blood flow, and increased coagulability, all of which are present in chronic venous disease [28]. In addition, varicose veins are a risk factor for both superficial vein thrombosis and deep vein thrombosis. Many additional risk factors and underlying pathologic conditions (e.g., malignancy, thrombophilia, and autoimmune disease) are also described in relation to idiopathic, migratory, or recurrent superficial vein thrombosis. Apart from chronic venous disease, obesity, older age, female gender, smoking, oral contraceptives, and hormone replacement therapy are other frequently recognized risk factors [27].

In general, superficial vein thrombosis is considered a benign, self-limited disease that can cause considerable discomfort and affect mobility in some patients. However, it could lead to more serious complications. Recent and accumulating evidence suggests that it is often associated with more serious forms of venous thromboembolism, including deep vein thrombosis and pulmonary embolism, being recognized as an important risk factor in these conditions [29]. Literature reports a high frequency of thromboembolic complications associated with chronic venous disease, which vary between 22 and 37% in the case of deep vein thrombosis and up to 33% in the case of pulmonary embolism, indicating the need for diagnostic approaches and broader therapeutic approaches to diagnose and treat these possible complications [30].

Effective treatment of peripheral vascular disorders is important not only for the resolution of local symptoms, but also to prevent the development of systemic conditions such as deep vein thrombosis.

Prevalence of superficial thrombophlebitis

The incidence of superficial vein thrombosis remains unclear but is believed to be higher than that of deep vein thrombosis, which is estimated to be approximately one per 1000 cases. Although age is not an independent risk factor, the incidence of other risk factors increases with age, making superficial thrombophlebitis more common in older people and more frequent in women (50-70%). However, complications are less likely in people over 60 years of age [31-33].

In general, venous insufficiency has a high prevalence in developed and industrialized countries, but prevalence estimates vary widely by study setting, geographic region, and methodology [32]. The Vein Consult program evaluated more than 91,000 subjects in several geographical regions and found a worldwide prevalence of clinically significant chronic venous disease of 60% [23]. One in six men and one in five women suffer from chronic venous disease; however, this number has clearly decreased in the last 20 years compared to previous epidemiological data, perhaps due to earlier and more effective treatment [34].

In a study on the prevalence of chronic venous disease in the western European population, carried out in the urban and rural areas of Bonn (Germany), in a large group of adult patients (3702), in which the chronic venous insufficiency, varicose veins, the CEAP (clinical signs, etiology, anatomical distribution, and pathophysiology) clinical classes and leg symptoms (heaviness, tightness, swelling, pain after standing or sitting, pain when walking, muscle cramps, itching and restless legs), were monitored, it was observed that 90.4% of the investigated population showed venous changes, 14.3% varices without any other sign of chronic venous disease and 59.1% had isolated telangiectatic or reticular venous capillaries [35].

Varicose veins are important signs to consider in chronic venous disease, they have an estimated prevalence between 5% and 30% in the adult population, with a predominance of women to men of 3 to 1, although some studies support a higher prevalence in men [23]. However, in some studies, the estimates of the prevalence of varicose veins are higher: from 1% to 73% in women and from 2% to 56% in men [1]. A large population study in the United Kingdom has shown an age-adjusted prevalence of 39.7% in men and 32.2% in women, although these require more frequent treatment [36]. The age of onset varies in general; thus, some people develop varicose veins in adolescence, but the prevalence increases according to genetic [37], epigenetic, cultural, dietary-nutritional, and age factors [4].

One-third of the world's population has centripetal lymphovenous insufficiency of the upper and lower extremities, and 1% of them have chronic, unhealed trophic ulcers. The treatment costs around $12,000 a month. Around 2.5 million Americans suffer from chronic venous insufficiency of the lower extremities; 500,000 of them have trophic ulcers, with a dramatic socioeconomic impact, not only social but also labor, estimated at 2 million working days per year [23].

Factors increasing the risk of chronic venous insufficiency

In general, venous disorders show a significant association with several leg symptoms. Itching, a feeling of heaviness or oppression in the afternoon, seems to be more closely related to the venous phenomenon than other symptoms, although they may also be associated with other personal characteristics such as older age, female gender, and obesity. It is well known that obesity is associated with many disorders, one of which is severe venous stasis disease. In fact, overweight patients (body mass index >25) and many severely obese patients (body mass index >40) have pitting pretibial edema, but some have refractory pretibial venous stasis ulcers or pretibial bronze edema as a result of erythrocyte extravasation in post-phlebitic syndrome. In fact, chronic venous insufficiency is the most common cause of leg ulcers, which implies that morbidly obese patients are characterized by particularly recalcitrant ulcers. Although patients with class III obesity have severe extremity symptoms typical of chronic venous insufficiency, approximately two-thirds of the extremities have no anatomic evidence of venous disease. In any case, the association between increased extremity symptoms and obesity suggests that obesity itself contributes to morbidity [38-40].

Not only the association between obesity and varicose veins is especially relevant in women, but the relationships between comorbidities and chronic venous disease seem to be different between men and women. A cross-sectional study with 1,679 patients (65.0% women) reported that mild forms of chronic venous disease were (11.6%) more frequent in women, while severe forms were more frequent in men (42.1%). On the other hand, the comorbidities found were emphysema and chronic obstructive pulmonary disease (COPD), which were equally present in both groups. However, women presented a higher frequency of diabetes mellitus, arterial hypertension, and skeletal/joint diseases than men [41].

Heparin Gel for the symptomatic management of uncomplicated superficial thrombophlebitis

Heparin 1000 IU/g Gel is a sodium heparin for topical use that was approved for the first time in Italy on July 27, 1972, in the pharmaceutical form of a gel containing 1000 IU of sodium heparin per gram [42].

The clinical efficacy of Heparin 1000 IU/g Gel focused on the treatment of venous disorders of the lower limbs, such as uncomplicated superficial thrombophlebitis, has been demonstrated in all phases of clinical development and post-marketing studies throughout several years (period from 1984 to 2001) with several clinical trials of phase II [7-10,43], phase III [11-13,19,21], and phase IV [14] and confirmed in clinical studies as well as analysis conducted after market authorization as topical sodium Heparin 1000 IU/g Gel, to treat superficial thrombophlebitis.

In the first set of clinical studies with this topical Heparin Gel, the primary objective of treatment was to improve the symptoms of uncomplicated superficial thrombophlebitis. It was demonstrated in multiple studies, one of which involved 31 patients diagnosed with superficial thrombophlebitis (15 subjects), hypodermitis (four subjects), and complications after sclerotherapy (12 subjects). Heparin 1000 IU/g Gel was applied for a period of four weeks, twice a day, spread over a zone of 3-10 cm long, depending on the degree of the condition. Clinical status was assessed at 14 days by monitoring particular symptoms, i.e., induration, pain, edema, and impaired limb function. In an overall evaluation with Heparin 1000 IU/g Gel, a large positive effect was observed in alleviating each monitored symptom. The acceptance of the preparation by the patients was very good. The patients positively evaluated the therapy with this preparation as an effective therapy, and at the same time, about half of them had the opportunity to compare it with other local heparin-containing preparations. None of the patients experienced further progression of inflammatory symptoms after starting local therapy with Heparin 1000 IU/g Gel. Most patients (78%) with advanced signs of chronic venous insufficiency grade II to III were followed up on an outpatient basis [15].

In a larger study including 412 patients with superficial phlebitis, a mean age of 53.1 and a mean weight of 77.8 kg, Heparin 1000 IU/g Gel was applied by massaging the gel into the skin with gentle movements, in an area 2 cm wide by 10 cm long. When the irritation disappeared, the patients were asked to apply the drug to an area that exceeded the size of the tender lesion by 1 cm. The application area was marked in such a way that it did not exceed 20 cm^2^. The use of elastic bandages and walking exercises was also recommended. The degree of patient satisfaction was assessed using a questionnaire to rate spontaneous pain, pain on palpation or pressure, ankle edema, extent of the skin rash, size and number of thrombophlebitic segments or nodules, and elevated body temperature with weekly controls. It was confirmed that Heparin 1000 IU/g Gel helps to control symptoms, reducing pain, sensitivity, and ankle edema. In 96% of cases, health professionals were satisfied with the good results of the therapy [16].

Phase III studies were conducted in other types of phlebitis, such as acute superficial phlebitis secondary to intravenous catheterization. The efficacy and tolerability of Heparin 1000 IU/g Gel was compared against placebo in a randomized, double-blind study of 126 patients with superficial thrombophlebitis after infusion. Gel or placebo was applied to the lesions three times a day until clinical remission, for a period of seven days. In the intention-to-treat analysis, Heparin 1000 IU/g Gel produced further healing than placebo (34.4% vs. 21.5%, respectively; p = 0.033), resulting in a relative advantage of healing in favor of Heparin 1000 IU/g Gel of 1.69 (95% CI: 1.03-2.78). The number of patients needed to treat (NNT) and achieve clinical healing was six (6). A withdrawal rate was observed in the treatment groups of 36.4% with Heparin 1000 IU/g Gel versus 37.9% with placebo, all of which were considered failures in the intention-to-treat analysis. The clinical evolution of phlebitis and the global perception of the researchers were in favor of the group treated with Heparin 1000 IU/g Gel. Only one adverse event was reported: mild contact urticaria in a patient treated with Heparin 1000 IU/g Gel that was considered probably related to treatment [17].

Studies were also conducted applying the same active pharmaceutical ingredient (topical heparin) as gel or hydrogel in doses ranging from 200 IU/g to 2400 IU/g, showing similar results to those obtained with Heparin 1000 IU/g Gel, and demonstrating that the use of topical Heparin Gels reduces the symptoms of superficial vein thrombosis [44,45]. In addition, this active pharmaceutical ingredient has been used as a topical formulation in other pathologies such as scar management, trauma, bruises, and superficial wounds, alone or in combination with other active ingredients [46,47].

Systematic reviews have examined in depth the use of topical applications of heparin formulations for the treatment of vascular disorders. Vecchio and Frisinghelli reviewed 20 studies with a total of 1055 patients where topical heparin formulations were compared with placebo, without an active comparator, or with subcutaneous heparins, in the treatment of superficial thrombophlebitis or venous insufficiency. The authors conclude that, even though some clinical studies were performed years ago, Heparin 1000 IU/g Gel was more effective than placebo in reducing the signs and symptoms of superficial thrombophlebitis. Consequently, topical heparin preparations may be helpful in alleviating the signs and symptoms of vascular disorders while improving microcirculation. There is evidence to suggest that Heparin 1000 IU/g Gel may be more effective than other topical preparations in treating these conditions, possibly due to the relatively high levels of heparin in the gel formulation [48]. In a most recent review by Di Nisio et al., several treatments for superficial thrombophlebitis treatments of 33 clinical studies involving 7296 patients with superficial thrombosis of the legs are analyzed, including fondaparinux; rivaroxaban; low molecular weight heparin; unfractionated heparin; non-steroidal anti-inflammatory drugs (NSAIDs); compression stockings; and topical, intramuscular, or intravenous treatment to surgical interventions such as thrombectomy or ligation. The authors concluded, on the basis of the reported data, that topical treatments alleviated local symptoms [49]. 

Heparin 1000 IU/g Gel: Efficacy and safety in clinical practice

The clinical efficacy of Heparin 1000 IU/g Gel was demonstrated in all phases of clinical development, as well as during the long period that it is being used in clinical practice (Table 1). Data from 16 phase II, III, and IV clinical studies conducted from 1983 to 2001 were reported and were mostly focused on the treatment of lower limb venous disorders such as uncomplicated superficial thrombophlebitis [7,8,11-22,50]. In general, the gel was applied two to three times daily for a maximum period of 30 days, in concentrations ranging from 800 to 5000 IU per application, depending on the extent of the disease. Therapeutic activity was evaluated subjectively by assessing several parameters: spontaneous and provoked pain, edema, functional limitation, sensation of heaviness, venous turgor, ulceration, paresthesia, cramps, erythema, and global skin disease. Laboratory parameters, including specific analysis of hemostasis, were analyzed prior to the completion of treatment. Systemic availability was shown to be negligible, although coagulation analysis performed in clinical studies found no demonstrable changes. 

No deaths related to the use of Heparin 1000 IU/g Gel or serious adverse events were reported throughout the development of the clinical program. In a total of 16 reported clinical studies, including post-marketing, in a population of more than 1500 patients; one patient was discontinued due to an allergic reaction presumed to be due to the standard treatment; one patient developed a sensitivity reaction that resolved on discontinuation; and one patient had to stop the treatment after only one day due to the appearance of erythema and itching at the gel application site. These symptoms decreased spontaneously after two days. In a post-marketing study that included 891 patients, 18 patients were withdrawn from the study (2% of the study population) due to unwanted effects [14].

With the aim to corroborate the efficacy and tolerance in a wider outpatient population observed in the specialist clinical practice, a post-marketing surveillance study was performed to evaluate the efficacy and safety of a Heparin preparation for topical use in 890 patients (269 men and 621 women) hospitalized for venous or traumatic pathology with a clear painful component accompanied by edema, reduction of the functional capacity and dystrophy signs of the cutaneous district [14]. The objective was to evaluate the anti-inflammatory effect of a sodium Heparin Gel in superficial venous disorders of the extremities and in post-traumatic diseases. Sodium Heparin 1000 IU/g Gel was applied topically one to three times a day for 14 days. The treatment could have been prolonged, according to the physician’s advice, in the most severe cases, up to four weeks. The efficacy of the treatment was assessed on the basis of the improvement in the painful symptoms as well as the other signs and manifestations associated with the disease being treated, according to a four-score scale. Moreover, laboratory tests were done to investigate the blood-clotting function, including prothrombin time, partial thromboplastin time, clotting time, and platelet counting. A very significant improvement was observed in all the efficacy variables: the percentage of patients with moderate to severe symptoms; at the end of the treatment, they showed a considerable reduction of edema (54.0% to 6.1%), spontaneous (54.3% to 3.1%), induced pain (73.3% to 3.8%), sense of heaviness (68.2% to 10.4%), and functional impotence (39.1% to 5.0%). Thirty-five patients (3.9%) exhibited the following side effects: pruritus (10), erythema (6), allergy (5), dermatitis (5), dry skin (4), burning (2), sensation of cold (2), and urticaria (1). In most of the cases, the side effects showed moderate (42.5%) or mild severity (40.0%). Eighteen patients withdrew from the study due to other unwanted effects. In the tests on the hemostatic function, clotting time, partial thromboplastin time, and prothrombin time, a slight increase was recorded at the completion of the treatment period, which resulted to be statistically significant for clotting time and partial thromboplastin time. However, these modifications, in any case within a normal range, can be considered insignificant from a clinical point of view. No statistically significant changes were observed in the platelet count.

An analysis of data on the safety of Heparin Gel (250 IU/g and 1000 IU/g) therapy in Poland was done with data obtained from monitoring of spontaneous reports of adverse effects of 1.436.496 units of 250 IU/g gel and 435.799 units of 1000 IU/g gel. In addition, data from a literature search in several databases were included. It was concluded that Heparin Gel is a safe medication that rarely causes adverse effects since no unknown side effects were reported in the previous five years of the analysis [6].

In summary (Table 1), all the results generated in the clinical studies on the therapeutic activity and the tolerance of Heparin 1000 IU/g Gel confirmed, during the development and in the studies conducted after the commercialization, the efficacy and safety of this gel formulation, and are consistent with what has been observed in clinical practice where, in addition, greater adherence to treatment has been observed.

Systemic absorption of Heparin 1000 IU/g Gel

Systemic absorption proved to be insignificant when evaluating the safety of Heparin 1000 IU/g Gel. In the experimental models of toxicological evaluation, the levels with a toxic effect could not be demonstrated with repeated doses for one month in rabbits or three months in rats and dogs, and even with doses much higher than the maximum recommended in humans, after application of topical heparin in the same way. Neither did the topical application of Heparin 1000 IU/g Gel show any adverse effects in pregnant animals of rats or rabbits, nor in their fetuses [51].

The absorption of heparin in local applications depends on the type of patient, the anatomical region, and the concentration of heparin. In a clinical study with 32 patients diagnosed with superficial thrombophlebitis, hypodermitis, and complications after sclerotherapy, Heparin 1000 IU/g Gel was applied over a period of four weeks twice daily in 3 to 10 cm length of extended gel, according to the degree of affliction. The drug is absorbed in the different layers of the skin as follows: stratum corneum (45.3%), deep epidermis (10.9%), and dermis (2.9%). After applying the gel to the skin, the absorption is accelerated and accumulates in the horny layer of the epidermis. A second application of the preparation in approximately 2.5 hours increases penetration into the deeper layers of the skin [15].

It has been reported that heparin on intact skin is minimally absorbed into the circulatory system, with an approximate bioavailability in the dermis of 0.7%, including the microvascular area (<0.1%). About 50% of the active substance remains on the skin surface and in the first two horny layers. Therefore, the systemic availability of heparin after topical application of Heparin Gel is negligible, with no effect on standard coagulation tests [52,53].

Local tolerance of Heparin 1000 IU/g Gel

The studies on the local tolerance of Heparin 1000 IU/g Gel in animal models, such as rabbits and guinea pigs, stated that the product only induces mild and transient erythema but excludes the induction of skin sensitization, severe eye irritation, as well as the possibility of inducing phototoxic or photosensitive allergic reactions [54-57].

Heparin 1000 IU/g Gel was shown to be well tolerated in all studies [6,14,17,44]. Few patients treated with Heparin 1000 IU/g Gel were withdrawn from the studies due to the appearance of adverse events; most of them were mild or moderate. The types of adverse events were similar in the reference treatment group and in the Heparin 1000 IU/g Gel group. The most frequently reported adverse events were erythema, excessive dryness of the skin, itching and redness of the skin, dermatitis, urticaria, or feeling cold.

Pharmacovigilance of medications with Heparin 1000 IU/g Gel

The post-marketing safety of Lioton® and/or Menaven 1000 IU/g Gel, brands under which Heparin Gel 1000 IU/g is marketed, has been sufficiently demonstrated since it is marketed in 45 countries, 14 of them in the European Union, indicated not only for the treatment of venous disorders of the lower limbs, such as uncomplicated superficial thrombophlebitis, but also for varicose syndromes and their complications, such as phlebothrombosis, thrombophlebitis, superficial periphlebitis, varicose ulcers, post-operative varicophlebitis, post-saphenectomy sequelae, trauma and bruises, local edema and infiltrates, subcutaneous hematoma and for traumatic disorders of the musculotendinous and capsuloligamentous systems [58,59]. Given its evident safety profile, Lioton® or Menaven® 1000 IU/g Gel holds the regulatory status of an over-the-counter product and is not subject to medical prescription in many countries [60].

## Conclusions

Pharmaceutical formulations containing sodium Heparin 1000 IU/g in Gel (Lioton® and Menaven®) are mainly indicated for the treatment of diseases of the superficial vena. After a long time of therapeutic use, there have been no reports of urgent safety restrictions, marketing authorization withdrawals, revocations or suspensions, inability to obtain or renew a marketing authorization, distribution restrictions, suspension of clinical studies for safety reasons, dose modifications, changes in the target population or indications, nor changes in the formulation for safety reasons. There is no new information available on potential adverse effects during pregnancy and lactation, nor information that modifies the benefit-risk analysis in relation to the use of heparin-containing medicinal products in pediatric populations. Consequently, heparin-containing medicinal products in pharmaceutical forms of topic gel can continue to be considered medicinal products with an adequate safety profile and a positive benefit-risk ratio.
